# Supplementary material for: Are Internet Information Sources Helpful for Adult Crohn’s Disease Patients Regarding Nutritional Advice?
Source: J Clin Med. 2024 May 11;13(10):2834. doi: 10.3390/jcm13102834 (PMC11121864; doi:10.3390/jcm13102834)
Supplement: Supplementary file 1 [file jcm-13-02834-s001.zip › jcm-2990536-supplementary.pdf]

Analysis system

As already mentioned, the second evaluation form is based on an analysis system by Liebl et al. Based on this analysis system, 17 general criteria and 6 formal criteria were defined in order to scientifically evaluate websites (Liebl et al. 2015).
In order to make the individual points easier for the experts to understand, the sources specified by Liebl were analysed, summarised and assigned to the individual criteria. A distinction is made between general and formal criteria:

General criteria

Criterion 1: Completeness
When facts are included in patient information, all information must be checked. The scientific evidence must also be checked.

Criterion 2: Expertise - professional competence
It is important to identify the author of the text and to describe him/her and his/her qualifications in more detail. It must be stated whether the author is a non-physician or a physician. If the author is a medical doctor, their speciality must be stated. Even if it is, for example, a patient's testimonial, the patient's qualification must be stated. In addition, all other sources must be stated so that it is clear whether it is a scientific study or a personal experience, for example.

Criterion 3: Explanation of objectives and target group
It is important that the website makes it clear right from the start which target group the content is aimed at, e.g. medical specialists or patients, and what exactly the aim of the website is. The following questions can help with the evaluation: Which target group is the website intended for? Is there a clear distinction between factual content and advertising? Are clear objectives stated?

Criterion 4: Achievement of this objective
A high-quality site will fulfil the stated objective and provide the information that was announced.

Criterion 5: Appropriate balance/neutrality
Any vendor interests must be made transparent to the reader. The reader must be informed about possible conflicts of interest. There should also be a declaration of editorial independence. Attention should be paid to whether the page was written from an objective or personal point of view. Is the information based on just one source or on several? Is the site already certified by an independent third party such as Hon or Afgis? Caution is advised if the site bases its information on examples of individual cases or if the information is presented in a sensationalist manner.

Criterion 6: Precision
The facts on which the patient information is based must be scientifically accurate. Scientific means of verification are validity and reliability. Are the statements scientifically proven?

Criterion 7: Relevance/serviceability
The information must be up-to-date and meaningful. However, it is particularly important that it is also relevant. Relevance means that the recommendations and information are helpful to the reader when making decisions on health issues. The pages should be fully focussed on the needs of the user and the advice offered should be realistic and appropriate.

Criterion 8: Intelligibility for laypersons
Information in technical language, as well as the latest scientific findings, must be clearly explained to patients so that they can correctly assess the advantages and disadvantages of therapies.

Criterion 9: Suitability to support shared decision making
The information on a website should be designed to support, but not replace, the existing doctor-patient relationship. Decision aids should always be evidence-based. It is good information if it empowers a patient to make decisions. The assessment should look for suggestions on relevant topics that patients can discuss with those around them.

Criterion 10: No statements on topics without scientific evidence/proof
Uncertainties and gaps in the scientific data must be described. For example, if the effect of a treatment method has not yet been scientifically proven, no significant advantage or disadvantage can be described or no clear result is yet available, then this must be clearly indicated.

Criterion 11: Scientific evidence and timeliness
Patient information on the subject of health must always be up to date. It must also be clear when a website was created and when it was last updated. It must also be stated when and if an upcoming update is planned. Sources are required for every statement and must always be provided in full.

Criterion 12: Information on additional resources and recommendations (benefits and risks, impact on quality of life, consequences of non-treatment)
The disease and its natural course should be described so that a patient knows what the course of the disease looks like without treatment. Reference should generally be made to supporting literature, as it is rarely possible to answer all questions at once. Sometimes counselling centres and self-help groups can provide a patient with additional support.

Criterion 13: Focus/emphasis/orientation on the patient
In order for a patient to benefit from facts and knowledge, the information must be relevant to them and they must be able to understand it. Comprehensible and linguistically adapted information is a prerequisite. The latest scientific findings must be checked individually for each patient with regard to the usefulness of treatment.

Criterion 14: Layout aspects
The website should be clearly laid out and patients should be able to find their way around easily. General information such as the table of contents should be accessible from each

individual HTML page and the homepage should be easy to reach. Font size 12 or 14 and a clear font should be used. The colour design should support a fluent comprehension of the content. Images can help to make the text easier to understand.

Criterion 15: Quality management
The HONcode is regarded as the standard for good science and ethical behaviour for the publication of medical information on the Internet. The HONcode certification stands for high-quality medical information. Certification means that website operators are obliged to comply with the quality assurance standards prescribed by the code. A website with such certification can be clearly recognised by the logo on the site. If a seal is awarded, it can be assumed that the health information has been checked and is of high quality. If a website is not certified by a third party, it must be clear who is responsible for the content of the website, the updating of the information and the sources.

Criterion 16: Clear organisation of information
A website must make it as easy as possible for the reader to recognise whether it is providing specialist information or advertising. It must be clear who is responsible for the website and its content and what kind of sources the publishers are referring to. In particular, it should be emphasised whether these are field reports or scientific studies and how high the level of evidence is.

Criterion 17: Labelling of missing scientific evidence and risks
In general, it must also be clear here whether the information is scientifically based health information or advertising. The authors must always state their qualifications. Risks and benefits must be stated for each recommendation. There should be clear indications if there are grey areas with regard to the state of scientific research. Such an unclear data situation exists, for example, if there is no evidence of the effectiveness of advice or forms of therapy or if these are contradictory. It should also be made clear if it is unclear who is most likely to be affected by benefits or risks. A high quality site will provide information if advice or a treatment option cannot be clearly recommended and therefore the impact on the patient is uncertain.

Attention should be paid to the occurrence of discussions regarding gaps in knowledge or differences in expert opinion regarding recommendations, and vigilance should be exercised when a site describes recommendations as one hundred per cent effective or the effects on every patient are described as the same.

Formal criteria

Formal criterion 1: Transparency regarding providers, supporters, funding, advertising
Firstly, it is important to state what type of information is involved and whether it is based on data from studies, the experience of experts or the opinions of individuals. It must also be

immediately clear whether it is editorial content or advertising. All promotional or advertising content must be clearly labelled with the word advertising. A site must also provide clear information on sponsorship and funding. In addition, any conflicts of interest must be indicated.

Formal criterion 2: Data protection
The provider must state whether and to what extent user data is used and stored and whether it is passed on to third parties. This also includes email addresses and email content between the provider and users. A privacy policy should provide information on these topics. Does it mention whether the website uses cookies?

Formal criterion 3: Completeness of information regarding sources of evidence
Sources must be cited for all medical and health-related information, unless it is a personal experience. Sources include databases, medical guidelines, patient guidelines, patient experiences and internet research. The author or originator cannot be accepted as the sole source. The citation must be complete and dated, and the information must always be based on the latest scientific findings.

Formal criterion 4: Compliance with scientific knowledge regarding numbers and outcomes
The aim of the website should be to provide only evidence-based information. The information must be as up to date as possible and include the latest scientific findings. The claim should be that every statement is based on scientific evidence.

Formal criterion 5: Language adapted to the needs of the target group
The current state of science should be converted into understandable information that can be easily grasped by the target group and is adapted to their needs. This requires the explanation of technical terms and a simple choice of words. A clear structure can also contribute to a better understanding of the content. Verbose explanations should be avoided.

Formal criterion 6: Possibilities of feedback and participation for users - Possibility of feedback and participation for users
A site must give visitors the opportunity to provide feedback on questions, suggestions, criticism and problems. There must be a contact person with an email address or a contact form.
